# Simulated Trends in Ionosphere‐Thermosphere Climate Due to Predicted Main Magnetic Field Changes From 2015 to 2065

**DOI:** 10.1029/2019JA027738

**Published:** 2020-03-13

**Authors:** I. Cnossen, A. Maute

**Affiliations:** ^1^ British Antarctic Survey Cambridge UK; ^2^ National Center for Atmospheric Research Boulder CO USA

**Keywords:** ionosphere, thermosphere, magnetic field, long‐term trend, secular variation, prediction

## Abstract

The strength and structure of the Earth's magnetic field is gradually changing. During the next 50 years the dipole moment is predicted to decrease by ∼3.5%, with the South Atlantic Anomaly expanding, deepening, and continuing to move westward, while the magnetic dip poles move northwestward. We used simulations with the Thermosphere‐Ionosphere‐Electrodynamics General Circulation Model to study how predicted changes in the magnetic field will affect the climate of the thermosphere‐ionosphere system from 2015 to 2065. The global mean neutral density in the thermosphere is expected to increase slightly, by up to 1% on average or up to 2% during geomagnetically disturbed conditions ( $K_{p}\geq 4$). This is due to an increase in Joule heating power, mainly in the Southern Hemisphere. Global mean changes in total electron content (TEC) range from −3% to +4%, depending on season and UT. However, regional changes can be much larger, up to about ±35% in the region of ∼45°S to 45°N and 110°W to 0°W during daytime. Changes in the vertical $\vec{E}\times \vec{B}$ drift are the most important driver of changes in TEC, although other plasma transport processes also play a role. A reduction in the low‐latitude upward $\vec{E}\times \vec{B}$ drift weakens the equatorial ionization anomaly in the longitude sector of ∼105–60°W, manifesting itself as a local increase in electron density over Jicamarca (12.0°S, 76.9°W). The predicted increase in neutral density associated with main magnetic field changes is very small compared to observed trends and other trend drivers, but the predicted changes in TEC could make a significant contribution to observationally detectable trends.

## Key Points

- Predicted magnetic field changes from 2015 to 2065 cause a very minor (up to 1–2%) increase in global mean thermosphere neutral density
- Effects on total electron content are larger, up to ±35% in the American sector ( ∼45°S to 45°N, 110°W to 0°W)
- Reduced vertical $\vec{E}\times \vec{B}$ drifts at Jicamarca and surrounding longitudes ( ∼105–60°W) cause a weakening of the equatorial ionization anomaly

## 1 Introduction

The strength and structure of the Earth's magnetic field is continually changing: Over the past ∼180 years, the magnetic dipole moment has decreased by about 10%, while the magnetic dip poles have been moving northwestward in both hemispheres (Jackson et al., 2000; Thébault et al., 2015). The strongest changes in the magnetic field have occurred over South America and the southern Atlantic Ocean due to the westward movement, expansion and deepening of the South Atlantic Anomaly (SAA), a region of weak magnetic field intensity. A recent prediction of the Earth's magnetic field by Aubert (2015) showed that these trends are expected to continue during the coming century.

The method used by Aubert (2015) combines geomagnetic data with a geodynamo model in a data assimilation framework to predict the geomagnetic field up to 100 years into the future. A single step of an ensemble Kalman filter is used to represent the spread of the observables, and the numerical integration of the ensemble using the prognostic equations produces a forecast. The ensemble spread gives an estimate of the prediction error. The 50‐year forecast starting in 1965 provides a reasonable representation of the real coefficient evolution, which gives some confidence in the 50‐year forecast into the future. By 2065 the SAA minimum is predicted to intensify by −1.46±0.4μT at the Earth's surface, to move westward by 12.8° ± 1.4°, and widen. The dipole moment decreases by about 3.5%, from $7.78\times 10^{22}$ Am ${}^{2}$ in 2015 to $7.51\times 10^{22}$ Am ${}^{2}$ in 2065.

The secular variation of the Earth's magnetic field has significant effects on the ionosphere‐thermosphere system (Cnossen & Richmond, 2008, 2013; Yue et al., 2008) and is one of the most important drivers of long‐term changes in the ionosphere (Cnossen, 2014), in addition to the increase in atmospheric CO ${}_{2}$ concentration. Most studies on long‐term changes in the upper atmosphere have focused on detecting and understanding the causes of past trends (e.g., Cnossen, 2012; Laštoviča, 2017; Qian et al., 2011, and references therein). However, for practical purposes, such as long‐term planning for new satellite missions, managing the risks of space debris, and assessing space weather impacts on space assets and infrastructure, predictions of future changes are needed.

In this study we examine how the ionosphere and thermosphere are expected to respond to future changes in the main magnetic field, according to the prediction by Aubert (2015). We will focus on climatological changes in neutral density and total electron content (TEC), including spatial variations and dependencies on season, time of day, and level of geomagnetic activity. Neutral density is an important parameter to consider due to its effect on satellite drag. The observed long‐term decline in thermosphere density (e.g., Emmert, 2015) reduces drag on active satellites and space debris, affecting orbital characteristics and lifetimes. It is important to understand what future changes can be expected for appropriate mission planning and managing the risks of the growing space debris population (e.g., Lewis et al., 2011). TEC is important for Global Navigation Satellite Systems signal propagation and applications. Any applications that require long‐term measurement stability (e.g., sensitive climate monitoring) require a good understanding of long‐term changes in TEC to avoid spurious long‐term signals in the data products (Scharroo & Smith, 2010). Long‐term changes in neutral density and TEC could therefore both have important practical implications. Local effects of magnetic field changes on the ionosphere are studied in more detail at the location of Jicamarca (12.0°S, 76.9°W). Jicamarca has a long data record of plasma density and drifts and has played a crucial role in studies of the low‐latitude ionosphere. It is therefore important to understand how changes in the magnetic location of the station over time will affect measurements made here and their interpretation.

## 2 Methodology

**Figure 1:**
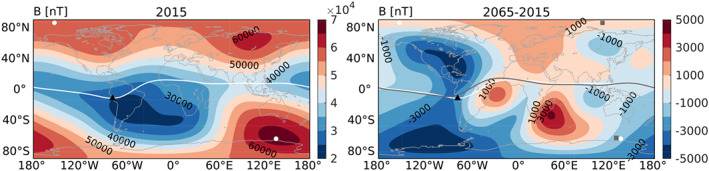
Magnetic field intensity (nT) in 2015 (left) and the difference between 2065 and 2015 (right) based on the IGRF (2015) and the Aubert (2015) prediction (2065). The white (gray) line marks the magnetic equator in 2015 (2065), and white dots (gray squares) mark the positions of the magnetic dip poles in 2015 (2065). The black triangle marks the location of Jicamarca, which we examine in section 3.3.

Simulations with the Thermosphere‐Ionosphere‐Electrodynamics General Circulation Model Version 2.0 (Richmond et al., 1992; Qian et al., 2014) were performed at a horizontal resolution of 2.5° × 2.5° and vertical resolution of 1/4 scale height, with vertical levels ranging from ∼97‐ up to ∼500‐km altitude. Two full‐year simulations were done: one with the magnetic field of 2015 as specified by the International Magnetic Reference Field 12 (Thébault et al., 2015) and one with the magnetic field of 2065 as predicted by Aubert (2015). We will refer to these as mf2015 and mf2065, respectively. We chose to simulate the year 2015 as a baseline, as it is a recent epoch and contains a mixture of more disturbed and quiet times, and its magnetic field is fully specified in International Magnetic Reference Field 12. The year 2065 was chosen as the future epoch to compare with 2015, as it is sufficiently far into the future to expect significant changes to occur, while the estimated error of the Aubert (2015) prediction is still relatively small. Figure 1 shows the predicted magnetic field changes between 2015 and 2065. As noted before, the SAA region moves westward and expands, with the magnetic field intensity continuing to decrease here, while the magnetic dip poles move northwestward. The dipole moment decreases by about 3.5%, but at high latitudes, the change in field intensity is larger in the Southern Hemisphere (SH) than in the Northern Hemisphere (NH).

Apart from the difference in the main magnetic field, the two simulations were set up identically. Both were run with observed solar and geomagnetic drivers for 2015. Solar wind and interplanetary magnetic field parameters from the OMNI data set were used to drive the auroral parameterization and the Weimer (2005) potential model, specifying the high‐latitude electric field. Both the aurora and high‐latitude electric field are specified on the magnetic grid, so that differences between the two simulations will occur on the geographic grid. Instantaneous and 81‐day average F10.7 data were used to characterize the solar radiative forcing. The climatological zonal mean lower boundary temperature and horizontal winds were set with the Naval Research Laboratory Mass Spectrometer Incoherent Scatter radar model (Picone et al., 2002) and the Horizontal Wind Model 2014 (Drob et al., 2015), respectively. Perturbations in the lower boundary temperature, horizontal wind, and geopotential height associated with diurnal and semidiurnal migrating tides were specified by the Global‐Scale Wave Model (Zhang et al., 2010). Nonmigrating tides were not included, as the Thermosphere‐Ionosphere‐Electrodynamics General Circulation Model Version 2.0 is not tuned for these, but we expect that this would only have a minor effect on our results. Each simulation was initialized from a 20‐day spin‐up simulation to allow the model to reach a quasi steady state. Full simulations were run from 0 UT on 1 January to 23 UT on 31 December, with hourly data stored for analysis.

While the model works with pressure coordinates, observations are normally done with reference to geometric altitude. We therefore interpolated model outputs to geometric height and analyze results as a function of height or at a constant height level. The effects of changes in the magnetic field on the climate of the ionosphere‐thermosphere system are estimated as the average difference between the simulations. To indicate whether these differences are significant in the light of typical day‐to‐day variability, we performed a Student's t test, where the standard deviation serves as a measure of the variability. For example, to assess the significance of the difference in a quantity at a given location at a given UT averaged over the full year (365 data points), the standard deviation over all those data points would be used as input to the t test. In the diagnostics we indicate differences that are significant at the 95% level with shaded contours, while line contours are used for nonsignificant differences. Standard deviations were similarly used to construct 95% confidence intervals, used in most of the line plots. In principle, these procedures indicate how likely it is that the differences between our simulations represent a change in the climate, that is, the average state of the ionosphere‐thermosphere system, rather than being caused by “weather”‐like variability. However, we note that the variability in the model is considerably underestimated due to the use of climatological lower boundary conditions, as well as high‐latitude forcings based on empirical models that do not capture all of the natural variability. In addition, the underlying assumptions of the t test that data points are independent and normally distributed are not fully met by the data, which can also result in inaccuracies in the significance testing, although independent testing with a bootstrapping method indicated that these inaccuracies were small. Still, significance estimates and 95% confidence intervals should be treated with a degree of caution.

## 3 Results

### 3.1 Neutral Density

**Figure 2:**
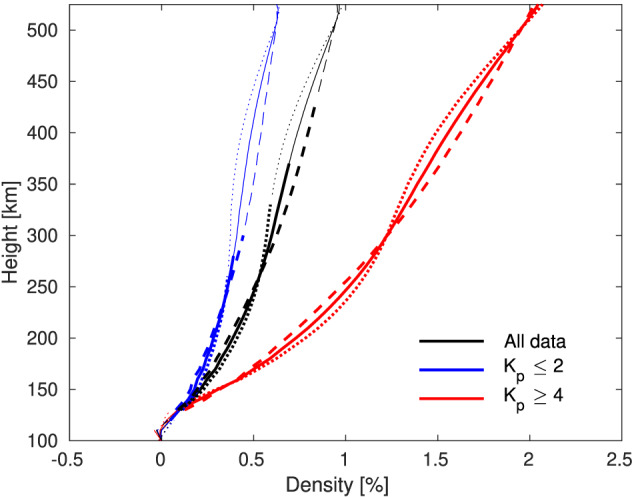
Difference (mf2065‐mf2015) in global mean (solid lines), NH (dashed lines), and SH (dotted lines) neutral density (%) versus height for the full year (black), for high geomagnetic activity conditions ( $K_{p}\geq 4$; red), and for low geomagnetic activity conditions ( $K_{p}\leq 2$; blue). Differences that are statistically significant at the 95% level are plotted with thick lines, while thin lines show nonsignificant differences.

The model results indicate that the neutral density in the thermosphere is almost everywhere larger for mf2065 than for mf2015. It therefore makes sense to examine the global and hemispheric mean differences in neutral density, as shown in figure 2. Global and hemispheric mean neutral density differences between mf2065 and mf2015 for three cases are shown: all of the data (average over the full year; 8,760 data points), low geomagnetic activity conditions ( $K_{p}\leq 2$; 4,550 data points), and high geomagnetic activity conditions ( $K_{p}\geq 4$; 816 data points). Differences are mostly small, with the maximum global mean difference for the full‐year average reaching only ∼1% and even less when geomagnetic activity is low. For high geomagnetic activity, the response more than doubles compared to the full‐year average, with the peak difference reaching just over 2%. Nonetheless, this is still a small change over a period of 50 years. There are small differences in the contributions from the NH and SH: The neutral density difference is consistently larger in the SH than in the NH in the lower thermosphere, up to ∼250‐ to 300‐km altitude, while the reverse is true in the upper thermosphere, up to ∼500‐km altitude. Dependencies on UT and season were found to be small and are therefore not shown.

The source of the increase in global mean neutral density is likely to be an increase in Joule heating, which causes the density at a fixed height in the thermosphere to increase due to thermal expansion. An increase in Joule heating is expected to occur in response to a reduction in main magnetic field strength (Cnossen et al., 2011, 2012; Wang et al., 2017), mainly due to enhanced ionospheric conductivity when the main magnetic field is weaker, while changes in neutral winds and ion convection can cause further modifications. Cnossen et al. (2012) found that the ionospheric conductivity scales on average approximately as $B^{-1.5}$, so that the ∼3.5% reduction in dipole moment from 2015 to 2065 is expected to lead to an increase in conductivity of ∼5%. We may therefore expect the Joule heating to increase by ∼5% as well, but spatial variations in changes in magnetic field strength, as well as changes in neutral winds and ion velocities, are likely to modify this figure. Cnossen et al. (2012) showed that a small decrease in magnetic field strength such as modeled here should cause an increase in $\vec{E}\times \vec{B}$ drift velocities, but since the Joule heating depends on the difference between ion and neutral velocities, it cannot be directly inferred that this should increase the Joule heating further. We also note that Cnossen et al. (2012) used simulations with a model that included the magnetosphere and therefore included effects of any changes in solar wind‐magnetosphere‐ionosphere coupling on the high‐latitude electric field, whereas the high‐latitude electric field in our simulations was prescribed, so that only effects of changes in the mapping between magnetic and geographic coordinates were included.

Table 1 confirms that the total Joule heating power is for the most part slightly higher for mf2065 than for mf2015, but the global mean difference for all data is a little smaller than expected, ∼3%. The difference is notably larger in the SH ( ∼6% for all data), which is consistent with high‐latitude changes in magnetic field strength being larger in the SH. It can also explain the slightly larger neutral density response in the SH below 250‐ to 300‐km altitude, as most of the Joule heating takes place in the lower thermosphere. The Joule heating differences are larger for high geomagnetic activity conditions, while they become very small or nonexistent for low geomagnetic activity conditions. This makes sense, as Joule heating is more important when geomagnetic activity is high, and it explains why differences in global mean neutral density are largest for high geomagnetic activity conditions. We further note that the Joule heating power is consistently larger in the NH than in the SH for mf2015, regardless of the geomagnetic activity level, while for mf2065 Joule heating power in the SH is larger, except for low geomagnetic activity conditions.

**Table 1:** Hemispherically and Globally Averaged Joule Heating Power (GW) for mf2015 and the Difference With mf2065 (mf2065‐mf2015) for All Data, High Geomagnetic Activity ( $K_{p}\;\geq \;4$), and Low Geomagnetic Activity ( $K_{p}\leq 2$)

	mf2015			mf2065‐mf2015		
	NH	SH	global	NH	SH	global
all	5.4	5.2	10.7	0.0 (0.0%)	**0.3 (6.2%)**	**0.3 (3.3%)**
$K_{p}\geq 4$	12.8	12.7	25.5	0.2 (1.3%)	**0.9 (7.4**%)	1.1 (4.4%)
$K_{p}\leq 2$	3.6	3.3	6.8	0.0 (0.0%)	**0.2 (5.2%)**	**0.2 (2.5%)**

*Note.* Differences that are significant at the 95% level according to a t test are printed in bold.

### 3.2 TEC

**Figure 3:**
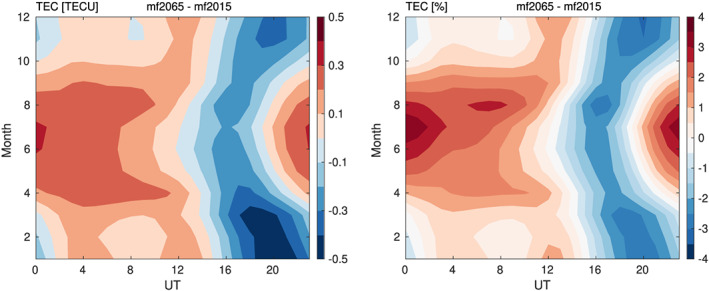
Difference in global mean TEC as a function of month and UT in TECU (left) and % (right).

Differences in global mean electron density between mf2015 and mf2065 depend considerably on season and UT. Most of the differences come from the $F_{2}$ peak electron density region and are clearly reflected in the TEC. Differences in global mean TEC as a function of season and UT are shown in Figure 3. The difference in global mean TEC is positive most of the time, peaking at just over 0.3 TECU (nearly 4%) around 0 UT in July. However, the TEC differences are predominantly negative between about 16 and 20 UT for all seasons, up to ∼−3% or nearly ‐0.5 TECU.

**Figure 4:**
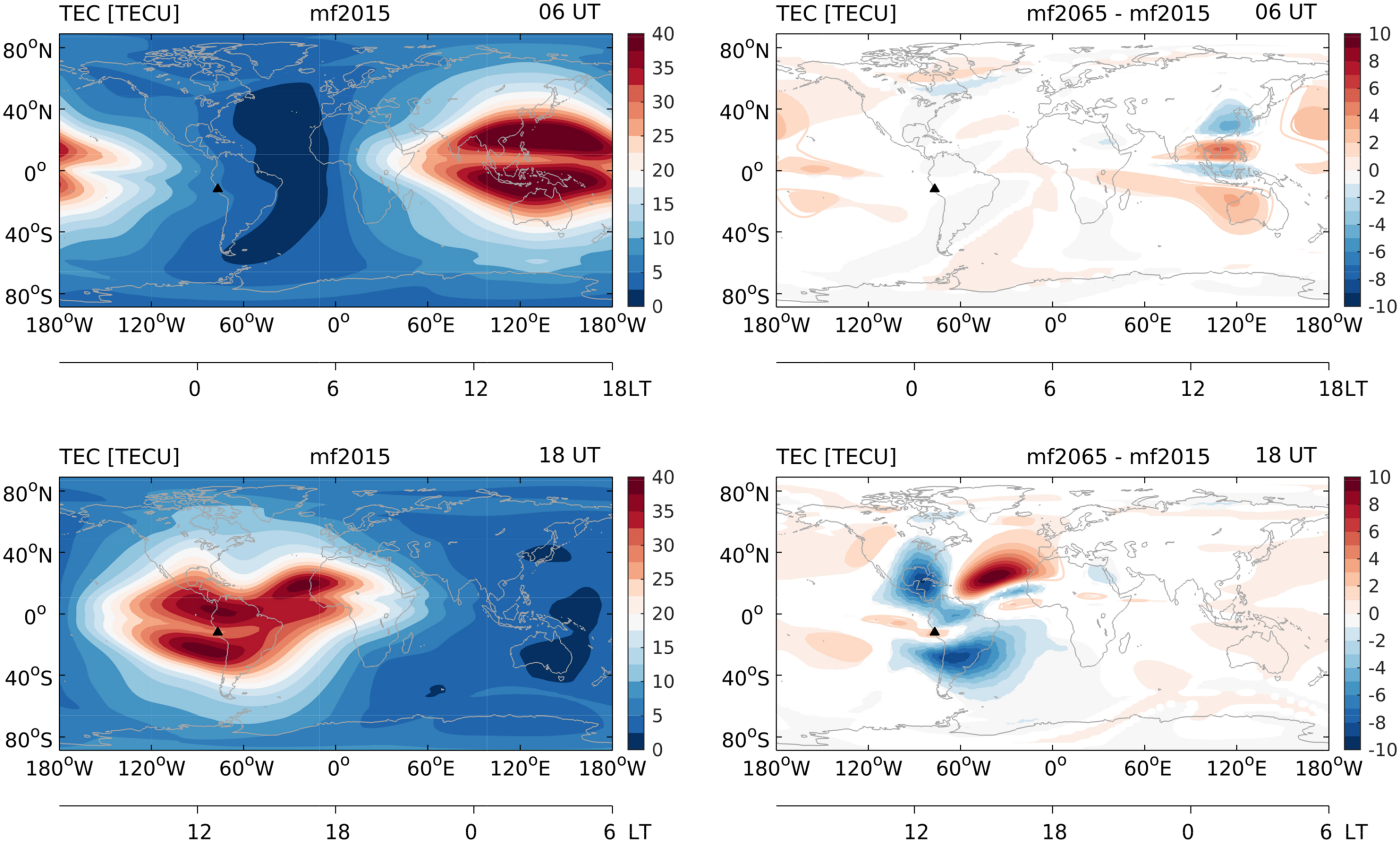
TEC (TECU) in 2015 (left) and the difference between 2065 and 2015 (right) at 6 UT (top) and 18 UT (bottom) averaged over all days of the year. The black triangle marks the location of Jicamarca.

Differences in TEC also vary considerably with location, as shown in Figure 4. The largest differences, of up to ±10 TECU ( ∼±35%), are found between about 110°W and 0°W from 45°S to 45°N, roughly in the region of the SAA, where predicted changes in the main magnetic field are relatively large. Large differences in TEC occur primarily during daytime, peaking around 17–19 UT, when it is afternoon in the SAA region. Since most of the response at that time is negative, this results in a negative global mean TEC difference, as shown in Figure 3. This contrasts with 6 UT, when there is a shift in the TEC pattern around 120°E, with the rest of the world mostly showing a small positive change. This illustrates that spatial variations in the TEC differences in combination with local time variations are responsible for the UT dependencies of the global mean TEC differences. TEC responses do not depend strongly on the geomagnetic activity level (not shown), which is probably due to the largest effects occurring at relatively low latitudes, where effects of geomagnetic activity are smaller than at higher latitudes.

**Figure 5:**
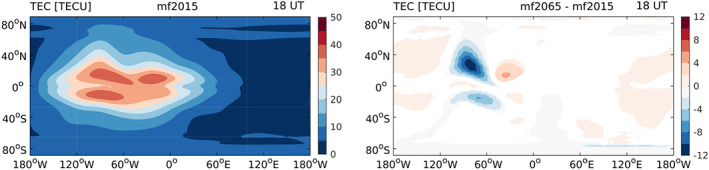
TEC (TECU) in 2015 (left) and the difference between 2065 and 2015 (right) at 18 UT plotted in magnetic latitude and geographic longitude averaged over all days of the year.

Since the TEC structure closely tracks the shape of the magnetic equator, differences in TEC can be expected to be related in part to the movement of the magnetic equator in a geographic coordinate frame, which changes the transformation between magnetic and geographic coordinates for the mf2015 and mf2065 cases. It is not possible to completely isolate the effect this has on the TEC, as the altered transformation has “real” physical effects due to the interaction of processes organized in geographic and magnetic reference frames. However, we provide an indication of the mapping effect by plotting the TEC for mf2015 and the difference with mf2065 at 18 UT as a function of magnetic latitude and geographic longitude in Figure 5. In the magnetic latitude frame the TEC shows less longitudinal structure and the TEC differences are considerably reduced in terms of their spatial coverage. On the other hand, the largest TEC response (in absolute terms) in this reference frame is nearly −12 TECU, while in a geographic reference frame this was no more than ±10 TECU.

Physically, changes in TEC can be driven by changes in plasma transport and/or by changes in composition. Plasma transport processes that are affected by changes in the magnetic field include $\vec{E}\times \vec{B}$ drifts, transport along the magnetic field induced by neutral winds, and ambipolar diffusion along the magnetic field (e.g., Cnossen & Richmond, 2012, 2013). The vertical component of these plasma transport processes in particular can drive changes in electron density: upward plasma transport into a regime of less recombination increases the plasma density, while downward transport acts to reduce the plasma density. Changes in the magnetic field can additionally effect the O/N ${}_{2}$ ratio through changes in circulation. For instance, an increase in Joule heating tends to lead to increased upwelling near the magnetic poles, which brings more molecular‐rich air up, reducing the O/N ${}_{2}$ ratio. A reduced O/N ${}_{2}$ ratio is associated with an increased recombination rate and thereby reduces the electron density.

**Figure 6:**
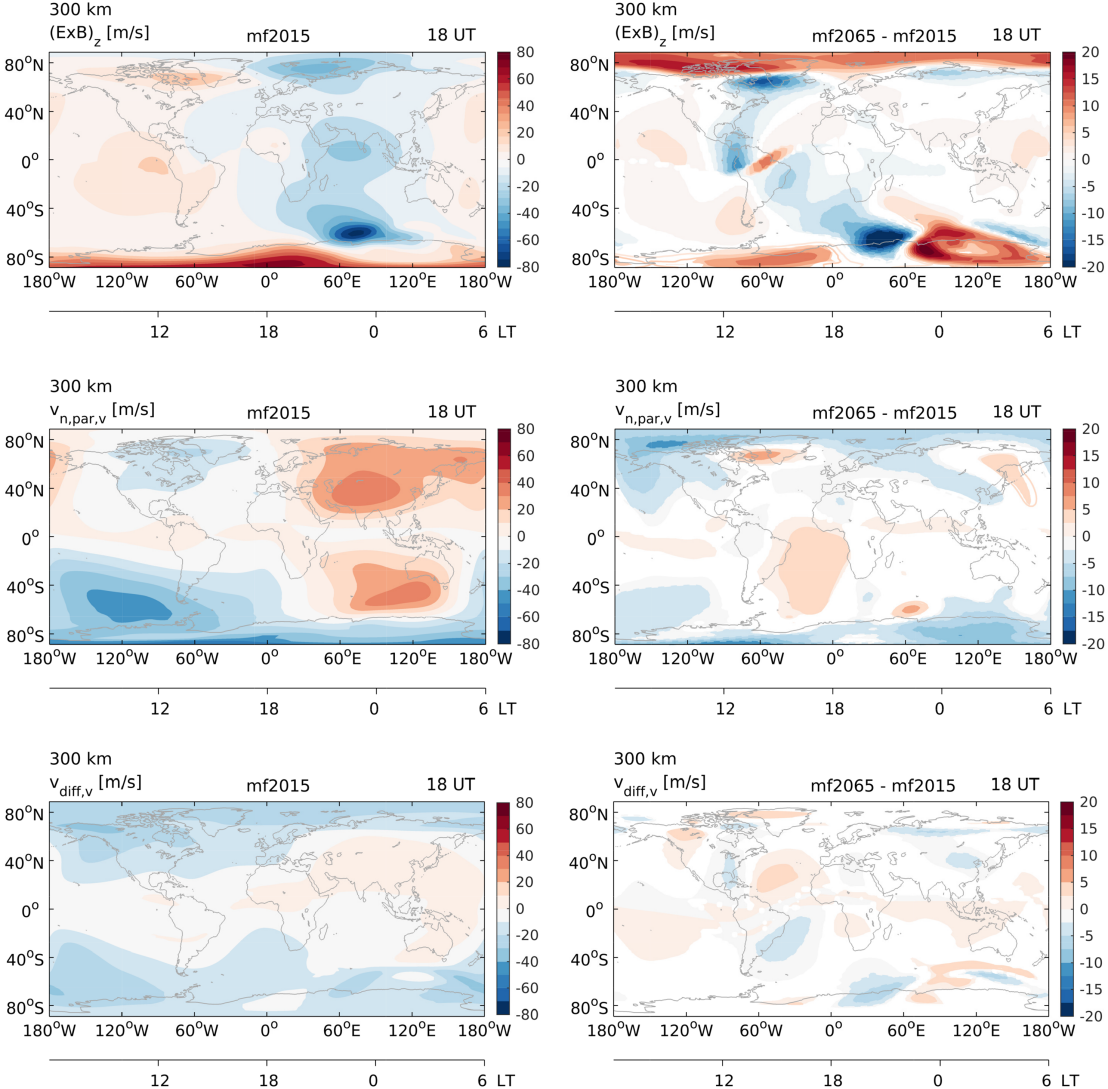
The vertical component of the $\vec{E}\times \vec{B}$ drift (top), the vertical component of the neutral wind projected onto the magnetic field ( $\left(\vec{U}\cdot \vec{b})\cdot (\vec{b}\cdot \vec{k}\right)$ (middle), and the vertical component of the field‐aligned diffusion (bottom) for mf2015 (left) and the mf2065‐mf2015 difference (right) at 18 UT averaged over all days of the year. Note that the color scale for the vertical $\vec{E}\times \vec{B}$ drift is fixed to ±80 m/s (mf2015; left) and ±20 m/s (mf2065‐mf2015; right) to allow for direct comparisons with the other velocity components and better visualization of the response at low to middle latitudes, while actual values at high latitudes are higher.

We find that significant changes in the O/N ${}_{2}$ ratio occur only at high latitudes (not shown) and are therefore unlikely to be responsible for the large low to midlatitude changes in TEC we see around 110–0°W at 18 UT. Figure 6 shows the vertical component of the $\vec{E}\times \vec{B}$ drift, the vertical component of the neutral wind projected onto the magnetic field (i.e., $\left(\vec{U}\cdot \vec{b})\cdot (\vec{b}\cdot \vec{k}\right)$, with $\vec{U}$ the neutral wind vector, $\vec{b}$ the unit vector along the magnetic field, and $\vec{k}$ the unit vector in the vertical direction), and the vertical component of the magnetic field‐aligned plasma diffusion at 18 UT for mf2015 (left) and the difference with 2065 (right), all at 300‐km altitude. The plasma diffusion was calculated under the assumption that O ${}^{+}$ is the dominant ion species, which is reasonable at this altitude. Comparison with Figure 4 (bottom) demonstrates that the mf2065‐mf2015 differences in the vertical component of the $\vec{E}\times \vec{B}$ drift and the vertical component of the field‐aligned plasma diffusion both have some similarities with the difference pattern in TEC, but there is not a direct one‐to‐one correspondence. The differences in vertical $\vec{E}\times \vec{B}$ drift are the largest out of the three variables shown here, and the differences are even larger in the UT hours preceding 18 UT (not shown). These prior changes also contribute to the TEC difference at 18 UT. Changes in the vertical $\vec{E}\times \vec{B}$ drift therefore seem to be the dominant cause of the changes in TEC around 110–0°W at 18 UT, although other plasma transport processes also play a role.

### 3.3 Jicamarca

**Figure 7:**
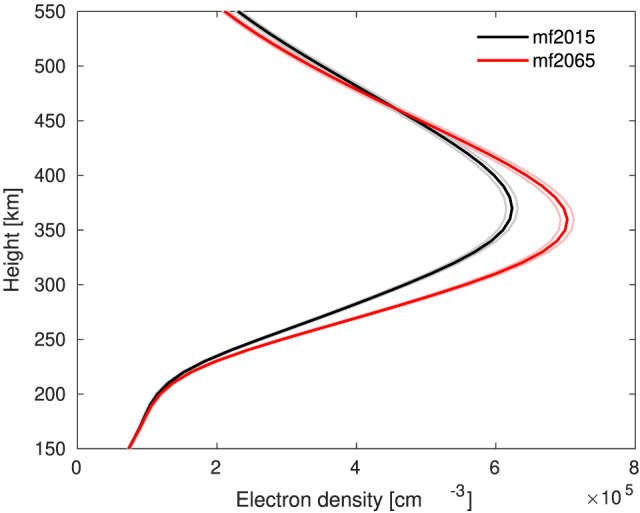
Annual mean electron density profile at Jicamarca for mf2015 (black) and mf2065 (red) averaged over all UTs. The 95% confidence interval is marked with thin lines.

To illustrate expected effects of future magnetic field changes on measurements from a widely used ground‐based station, we show several diagnostics for the location of Jicamarca (12.0°S, 76.9°W). This station is located within the region where magnetic field changes and their effects are relatively large, but as shown in Figure 4, it sits just in between two patches of strong reductions in TEC, where a local increase in TEC is found. Figure 7 shows the annual mean electron density profile over Jicamarca for the mf2015 and mf2065 cases averaged over all UTs. This confirms, as stated above, that most of the TEC changes come from the peak electron density region. In this case the peak electron density shows a significant increase, together with a slight reduction in the peak height.

**Figure 8:**
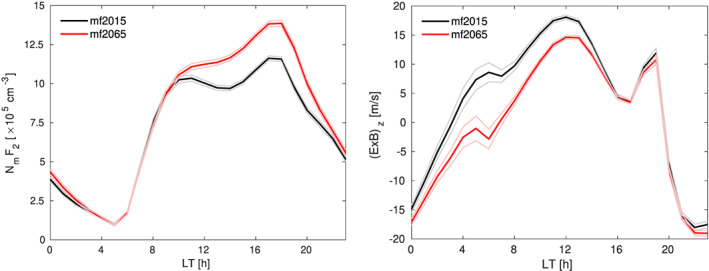
Annual mean $N_{m}F_{2}$ and vertical $\vec{E}\times \vec{B}$ drift at Jicamarca as a function of LT for mf2015 (black) and mf2065 (red). The 95% confidence interval is marked with thin lines.

**Figure 9:**
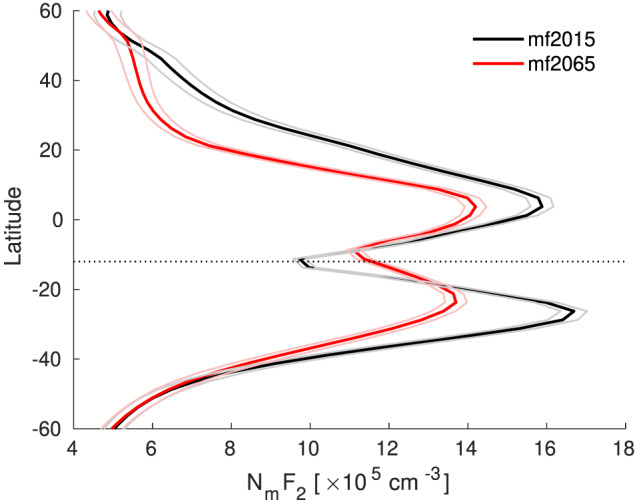
Annual mean $N_{m}F_{2}$ at 77.5°W and 18 UT (13 LT) as a function of latitude for mf2015 (black) and mf2065 (red). The 95% confidence interval is marked with thin lines. The black dotted line marks the latitude of Jicamarca.

Figure 8 shows the annual mean changes in the peak electron density, $N_{m}F_{2}$, and the vertical $\vec{E}\times \vec{B}$ drift at 300‐km altitude as a function of local time. This shows again that significant electron density differences occur primarily during daytime, although some remaining differences persist into the evening. At first sight it might seem that the differences in the vertical $\vec{E}\times \vec{B}$ drift do not correspond with the differences in $N_{m}F_{2}$ over Jicamarca, even though we identified this as an important mechanism in the previous section. However, this can be explained by the somewhat unusual positioning of Jicamarca. In 2015, it was located exactly on the magnetic equator, while the magnetic equator is expected to be located 4.3° northward of Jicamarca in 2065. For both cases this means that Jicamarca is located within the trough region of the equatorial ionization anomaly (EIA), although the station is slowly moving into the crest region. Figure 9 demonstrates that the shift of the EIA structure with respect to the station explains a small part of the local electron density increase. However, Figure 9 also shows that the more important effect is that the entire EIA structure in the longitude sector of Jicamarca becomes less pronounced for mf2065 than for mf2015, including a less pronounced trough. The weakening of the EIA structure is fully consistent with the reduction in the upward $\vec{E}\times \vec{B}$ drift we find at Jicamarca, as the EIA itself is driven by the low‐latitude vertical $\vec{E}\times \vec{B}$ drift (e.g., Anderson, 1981). Weaker vertical $\vec{E}\times \vec{B}$ drifts in the equatorial region therefore lead to a weaker EIA. Figure 4 indicates that the EIA at 18 UT is weakened throughout the longitude sector of ∼105–60°W. We also note that the more general statement made earlier that a reduction in magnetic field strength tends to increase the $\vec{E}\times \vec{B}$ drift velocities is not valid in the equatorial region due to large changes in the local electric field.

## 4 Discussion

Based on a large volume of satellite orbit data, Emmert (2015) showed that the global mean neutral density trend at 400‐km altitude is −2.0 ± 0.5% per decade between 1967 and 2005, while model simulations indicate that the increase in atmospheric CO ${}_{2}$ concentration causes a trend of up to −1.7% per decade at 400‐km altitude (Solomon et al., 2019). The increase in global mean neutral density of up to 1–2% we predict to result from changes in the main magnetic field between 2015 and 2065 translates to <0.5% per decade. It is therefore within the margin of error of what can be detected with observations and also around 5 times smaller than the estimated effect of past increases in CO ${}_{2}$ concentration. We therefore conclude that changes in the main magnetic field will at most have a very minor effect on long‐term changes in global mean density in the thermosphere over the period 2015–2065.

Changes in the main magnetic field have a more important effect on the electron density, as indicated by responses in TEC. Our analysis shows regional changes in TEC over a 50‐year period of up to ±10 TECU, corresponding to up to ±35%. This translates to up to ±2 TECU per decade or ±7% per decade. Such changes should be detectable observationally and could have implications for Global Navigation Satellite Systems signal propagation and applications. However, globally averaged trends are much smaller (up to 4% over 50 years, or 0.8% per decade) and depend on season and UT. They would be smaller still, if these dependencies were averaged out.

Observed trends in TEC for past epochs are somewhat unclear. Lean et al. (2011) reported a positive trend in TEC of 0.6±0.3 TECU between 1995 and 2010, but Laštoviča et al. (2017) stated there were several problems with their analysis, including an error in the underlying database. They argued there was no clearly detectable trend in TEC and a longer data set should be used. Still, Emmert et al. (2017) found that there was a change in TEC of −9.3% between the solar minima of 1996 and 2008 that could not be attributed to differences in the F10.7 and $K_{p}$ indices of solar and geomagnetic activity.

Recent modeling studies by Solomon et al. (2018) and Solomon et al. (2019) found that the increase in CO ${}_{2}$ concentration between the 1972–1976 and 2001–2005 periods caused a decrease in global mean $N_{m}F_{2}$ of 1.2% per decade. Changes in global mean TEC would probably be somewhat smaller than this, as the $F_{2}$ peak is the part of the ionosphere that shows the largest response to CO ${}_{2}$ changes. Our predicted 0.8% per decade change in global mean TEC due to magnetic field changes is then likely to be comparable in magnitude to the effect of the increasing CO ${}_{2}$ concentration. However, the two effects act in opposite directions for most UTs, which should reduce actually observed global mean TEC trends. The extent to which the two effects will cancel out, or indeed amplify each other, depends strongly on how data are averaged in space and time. A detailed comparison between observations and model results that takes into account dependencies on location, UT, and season is needed to determine how much of observed trends could be explained by the increase in CO ${}_{2}$ and main magnetic field changes combined, and by extension, to predict more precisely what will happen in the future. In this light it is also important to monitor TEC closely, especially in the region where long‐term trends associated with main magnetic field changes are expected to be large, that is, the region of ∼45°S to 45°N and 110–0°W.

While the present study was focused on climatic changes in the ionosphere‐thermosphere system, the dependence of some of the differences between the mf2015 and mf2065 simulations on the geomagnetic activity level indicates that the response of the system to geomagnetic storms may also change in the future. Larger Joule heating and neutral density differences for higher geomagnetic activity suggest that the ionosphere and thermosphere may become more severely impacted by a given disturbance in the solar wind as a result of main magnetic field changes. The Joule heating results presented in table 1 indicate that especially the SH will become more sensitive to geomagnetic activity increases. A et al. (2012) showed that neutral density responses to geomagnetic storms are already noticeably stronger in the SH than in the NH. Our results suggest that this asymmetry will become more pronounced in the future, although a more detailed analysis of the impact of main magnetic field changes on storm time responses should be done to confirm this. This will be the subject of a further study.

## 5 Conclusions

Predicted changes in the Earth's main magnetic field from 2015 to 2065 are expected to cause a small increase in global mean Joule heating of ∼2–4%, mainly coming from the contribution of the SH. The increased Joule heating causes a minor increase in global mean density in the thermosphere of up to 1% on average or up to 2% for geomagnetically disturbed conditions ( $K_{p}\geq 4$). Even taking the larger figure of 2%, this translates to a long‐term trend of <0.5% per decade, which is too small to be reliably detected with observations and also about 5 times smaller than the estimated effect of past increases in CO ${}_{2}$ concentration. Future changes in the magnetic field are therefore not expected to be a significant contributor to long‐term changes in thermosphere neutral density.

Predicted changes in the main magnetic field have a larger, more important effect on the ionospheric electron density, particularly in the region of ∼45°S to 45°N and 110–0°W, where main field changes are relatively large. In this region we find changes in TEC of up to ±10 TECU, corresponding to up to ±35%, during daytime. During nighttime, and in other geographical regions, changes in TEC are much smaller. Changes in the vertical $\vec{E}\times \vec{B}$ drift appear to be the most important driver of changes in TEC, but changes in other plasma transport processes also play a role. In the longitude sector of Jicamarca, a decrease in the low‐latitude vertical $\vec{E}\times \vec{B}$ drift causes a weakening of the EIA, leading to a local increase in electron density over Jicamarca, which is located within the EIA trough region. Global mean changes in TEC range from −3% to +4%, depending on season and UT. The predicted changes in TEC should be observationally detectable and could make a significant contribution to long‐term changes in TEC, depending on how data are averaged spatially and temporally.
